# The Reliable Detection of Homocysteine Using a Biosensor Based on Recombinant Cystathionine β-Synthase and Nanoporous Gold

**DOI:** 10.3390/microorganisms13030559

**Published:** 2025-03-01

**Authors:** Zihan Huang, Yan Gao, Lei Zhang, Ting Cai, Ruijun Liu, Xia Wang

**Affiliations:** State Key Laboratory of Microbial Technology, Shandong University, Qingdao 266237, China; hzh20212022@163.com (Z.H.); ygaosdu@163.com (Y.G.); leizhang10050304@163.com (L.Z.); cait2023@163.com (T.C.); 202332593@mail.sdu.edu.cn (R.L.)

**Keywords:** cystathionine β-synthase, nanoporous gold, homocysteine, electrochemical sensor

## Abstract

Given the essential roles of homocysteine (Hcy) and the interference of cysteine in effectively monitoring human health, this study proposed a synergistic effect strategy that combines the unique structural and functional properties of nanoporous gold (NPG) with the selective recognition capability of a recombinant cystathionine β-synthase (CBS) for the sensitive and specific detection of Hcy. The CBS protein with specific catalytic activity for Hcy was successfully produced in recombinant *Escherichia coli* BL21 (pET-30a-*cbs*) using the *cbs* gene from *Pseudomonas aeruginosa* PAO1. The electrochemical mechanism demonstrated that the electrooxidation of H_2_S, a catalytic product of the CBS, was an irreversibly surface-controlled process on the CBS/NPG/GCE electrode surface. The electrochemical detection of Hcy exhibited excellent linearity, with a high sensitivity reaching 10.43 µA mM^−1^ cm^−2^ and a low detection limit of 1.31 µM. Furthermore, the CBS/NPG/GCE biosensor was successfully used to detect Hcy in urine samples with strong anti-interference capability and high selectivity (relative standard deviation less than 2.81%), while effectively reducing the interference from cysteine. These results confirmed that the proposed CBS/NPG/GCE electrochemical sensor achieved specific, sensitive, and reliable rapid detection of homocysteine, making it highly promising for practical applications in clinical treatment and health assessment.

## 1. Introduction

Homocysteine (Hcy), a sulfur-containing amino acid not involved in protein construction, serves as a pivotal biochemical link connecting methionine metabolism with cysteine (Cys) biosynthesis [1,2]. Disturbances in Hcy metabolism result in an imbalance of redox states, accompanied by heightened oxidative stress and the generation of reactive oxygen and nitrogen species, which subsequently induce the oxidation of proteins, nucleic acids, and carbohydrates, along with lipid peroxidation, thereby constituting a significant hazard to human health [3]. Owing to its extensive biological roles in humans, accumulating research evidence has highlighted that fluctuations in Hcy concentrations within human plasma serve as a crucial marker for assessing overall health status [4]. An abnormal increase in plasma Hcy levels, termed hyperhomocysteinemia (HHcy), is regarded as cytotoxic and has been linked to various health complications, including cancer, neurological diseases, gastrointestinal conditions, chronic renal diseases, osteoporosis, and the development of congenital defects [5,6,7,8,9]. For example, digestive tract cancers, including gastric, esophageal, and colorectal cancers, account for approximately 22% of cancer-related deaths worldwide each year. A recent meta-analysis revealed that a 5 μM increase in Hcy levels correlates with a 7% higher risk of developing digestive cancers, suggesting that Hcy could serve as a potential biomarker for this category of cancer [4]. Furthermore, numerous studies have consistently shown that HHcy is a significant and independent risk factor for Alzheimer’s disease, the most common form of dementia. Specifically, every 5 μM increase in blood Hcy is directly associated with a 15% rise in the relative risk of developing Alzheimer-type dementia [10]. With the continuous advancements in the medical and healthcare fields, the increasing importance of Hcy in health and disease has garnered significant interest in its detection, which is essential for effectively monitoring human health.

Owing to the critical role of Hcy in clinical diagnostics, various conventional techniques have been developed for its detection, which can be categorized based on their underlying principles, including chromatography [11,12,13,14], enzyme cycling assays [15], fluorescence polarization immunoassay (FPIA) [16,17], and capillary electrophoresis (CE) [18,19]. While these established techniques ensure accuracy and sensitivity for Hcy detection, they are often accompanied by limitations such as prolonged procedures, high costs of reagents, labor-intensive sample preparation, and reliance on advanced instrumentation [20]. In contrast, electrochemical technology addresses the limitations of traditional detection methods, offering advantages such as ease of operation, exceptional sensitivity, rapid detection, and cost-effectiveness [21]. For instance, a previous study introduced a glassy carbon electrode coated with a composite film of Nafion and TiO_2_-graphene (TiO_2_-GR) nanocomposite for the rapid, straightforward, and sensitive voltammetric detection of the amino acid L-tyrosine (Tyr), achieving a sensitivity of 22.8 μA mM^−1^, with a detection limit as low as 2.3 μM, all within a relatively short timeframe [22]. Additionally, while fluorescent sensors and thermal imaging sensors are commonly employed for biochemical detection, electrochemical sensors offer several advantages over these methods. Unlike fluorescent sensors, which are limited by quenching effects, and thermal imaging sensors, which suffer from signal dissipation, electrochemical sensors do not rely on optical properties, which allows them to avoid interference from light absorption or scattering, and enable the easy acquisition of electrical signals that are minimally influenced by ambient thermal noise, thus ensuring stable and reliable sensor performance [23,24]. However, the intricate composition of human body fluids and the presence of numerous interfering substances have significantly constrained the extensive application of electrochemical sensor technology in medical diagnostics [25,26]. In addition, the coexistence of Hcy and Cys in body fluids, coupled with their remarkably similar structures and properties, poses a persistent challenge for researchers in designing Hcy-specific sensors [27].

Therefore, it is crucial to develop electrochemical sensors for the specific detection of Hcy, with strong anti-interference capabilities and applicability to real samples, while also ensuring excellent sensitivity and detection limits that meet practical requirements. Cystathionine β-synthase (CBS, EC 4.2.1.22), a crucial pyridoxal 5′-phosphate-dependent enzyme in the methionine cycle, primarily catalyzes the conversion of Cys and Hcy to cystathionine and H_2_S, as described in the literature [28,29]. Its high specificity for these substrates (Hcy and Cys) positions CBS as an ideal biorecognition element for electrochemical sensors targeting Hcy detection, effectively minimizing the interference from cysteine. In addition, nanomaterials have increasingly become a focal point in the development of recognition elements for electrochemical sensors [30,31], owing to their high specific surface area, excellent catalytic performance, and versatility in modification [32]. In particular, nanoporous gold (NPG), with its sponge-like structure, has gained recognition as a promising support for enzyme immobilization in bio-electrochemical applications, such as biosensors and biofuel cells, offering enhanced response and stability [33,34,35]. NPG-based sensors have demonstrated exceptional sensitivity and selectivity for a wide range of analytes from small molecules to biomolecules, including single-molecule detection [36,37].

**Scheme 1:**
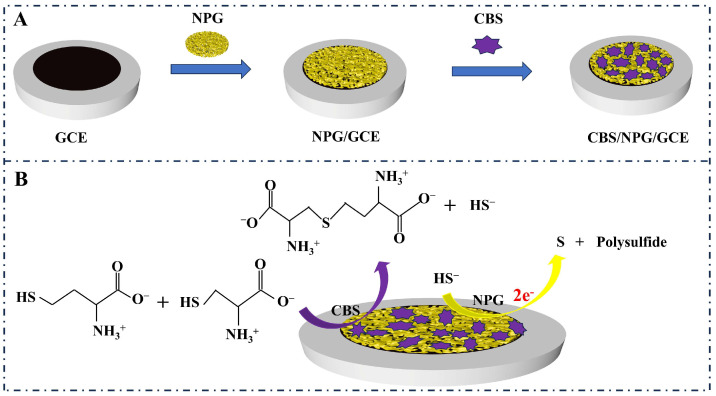
Schematic diagram of the construction for CBS/NPG/GCE electrode (**A**) and the principle of Hcy detection (**B**).

In this study, the CBS protein was heterologously expressed in a prokaryotic system using the *cbs* gene from *Pseudomonas aeruginosa* PAO1. Leveraging the unique structural and functional properties of NPG and the selective recognition capability of CBS protein, a reliable and highly sensitive CBS/NPG/GCE sensor for Hcy detection was successfully fabricated (Scheme 1A), where NPG served as an immobilization platform for the enzyme and CBS protein functioned as the biorecognition element, as shown in Scheme 1A. Based on this foundation, the principle of Hcy detection and the sensing capability of the CBS/NPG/GCE electrode constructed in this study were investigated. Additionally, the practical application potential of the CBS/NPG/GCE sensor was assessed through detailed anti-interference and spiked recovery experiments.

## 2. Materials and Methods

### 2.1. Reagents and Materials

L-homocysteine and tris(2-carboxyethyl) phosphine hydrochloride were obtained from Maclean Biochemical Technology Co., Ltd. (Shanghai, China). Creatinine was sourced from Beijing Solarbio Science & Technology Co., Ltd. (Beijing, China). Lead acetate test strips were purchased from Shanghai SSS Reagent Co., Ltd. (Shanghai, China), while nickel column packing (Ni-NTA) was obtained from Nanjing Kingsley Biotechnology Co., Ltd. (Nanjing, China). Additionally, alumina powder was sourced from Tianjin Aida Hengsheng Technology Development Co., Ltd. (Tianjin, China). High-purity nitrogen (purity > 99.9%) was supplied by Qingdao Benyi Gas Co., Ltd. (Qingdao, China). Methanol (chromatographic purity) and other analytical-grade chemical reagents were purchased from Sinopharm Chemical Reagent Co., Ltd. (Shanghai, China). Reagents and buffers were prepared using ultrapure water (ρ > 18 MΩ cm) from the millipore direct-Q3 UV machine.

To prepare a 0.5 M dilute sulfuric acid solution, dissolve the appropriate amount of concentrated sulfuric acid in ultrapure water. For the Nafion solution, weigh the required amount of 5% (wt%) Nafion solution (purchased from Kunshan Sunlaite New Energy Technology Co., Ltd., Kunshan, China), and dilute it with anhydrous ethanol to obtain a 0.5% (wt%) Nafion solution, which is stored away from light. To prepare the Luria-Bertani (LB) broth liquid medium, dissolve 10 g L^−1^ of tryptone, 5 g L^−1^ of yeast extract, and 10 g L^−1^ of sodium chloride in distilled water. Additionally, a phosphate-buffer solution (PBS, 50 mM, pH 8.0), prepared using Na_2_HPO_4_∙12H_2_O and NaH_2_PO_4_∙2H_2_O, was utilized as a supporting electrolyte. The lysis elution (LE) buffer (pH 8.0) contained 30 mM NaH_2_PO_4_ and 1 M NaCl.

### 2.2. Heterologous Expression of the CBS Protein

#### 2.2.1. Construction of the Recombinant Expression Strain

The *cbs* gene from *Pseudomonas aeruginosa* PAO1, a strain preserved in Prof. Luying Xun’s laboratory, was used as the template for primer design in polymerase chain reaction (PCR) amplification. The upstream primer (*cbs*-NdeI-F): GGAATTCCATATGATGACTTCCGACTCACGTCCCG and the downstream primer (*cbs*-XhoI-R): CCGCTCGAGTGCGAGCTTTCTCCGCAAATGGTTC were designed and the optimized PCR conditions were described in Table 1. The resulting PCR products were recovered by gel cutting and ligated with the pEASY-Blunt cloning vector. The ligated pEASY-Blunt-*cbs* recombinant plasmid was then transferred into the cloning host *Escherichia coli* DH5α for recovery culture, followed by plating the culture onto LB agar plates containing isopropyl β-D-1-thiogalactopyranoside (IPTG), X-Gal, and ampicillin for blue–white spot screening.

A single white colony selected from the blue–white spot screening was cultured in a liquid LB medium, and PCR amplification followed by sequencing was performed to confirm the presence of the positive clone. The pEASY-Blunt-*cbs* plasmid was double-digested with NdeI and XhoI restriction enzymes, and the resulting fragments were analyzed by 1% (*w*/*v*) agarose gel electrophoresis. Subsequently, the purified fragment was ligated into the similarly treated pET-30a expression vector and introduced into the *Escherichia coli* BL21 (DE3) host. Positive clones were selected on Kanamycin-resistant plates and further confirmed by PCR and double digestion for subsequent experiments.

#### 2.2.2. Expression, Purification, and Validation of Enzyme Activity

The recombinant strain was inoculated into a 1 L LB shake flask containing 50 μg mL^−1^ kanamycin, and cultured at 37 °C with shaking at 200 rpm for approximately 1 h until the optical density at 600 nm (OD_600_) reached between 0.6 and 0.8. Following this, IPTG was added to a final concentration of 0.5 mM, and the culture was induced at 25 °C with shaking at 200 rpm for 12 h. After induction, the bacterial culture was collected by centrifugation at 8000 rpm for 10 min, washed with LE buffer, resuspended in an appropriate amount of LE buffer, and then subjected to ultrasonic disruption. The disruption solution was then centrifuged at 4 °C (12,000 rpm for 10 min) to collect the crude enzyme solution containing CBS protein, which was subsequently purified using nickel column chromatography and eluted with LE buffer containing 250 mM imidazole to obtain the target protein. The imidazole solution containing CBS protein was desalted using an ultrafiltration tube, exchanged into PBS (50 mM, pH 8.0) buffer, and concentrated to 5 mg mL^−1^ using the same ultrafiltration method. For protein electrophoresis analysis, samples from the *E. coli* BL21 (DE3) crude enzyme solution, CBS protein-containing crude enzyme solution, and pure CBS enzyme solution were prepared. To confirm the catalytic activity of CBS protein, which can convert Hcy and Cys into H_2_S and cystathionine, a qualitative detection was performed using lead acetate test strips. In this experiment, 5 mM of Hcy and 5 mM of Cys substrates in PBS (50 mM, pH 8.0) were incubated with the enzyme solutions for 10 min, and the production of H_2_S was monitored using the lead acetate test strips clamped in Eppendorf tubes.

#### 2.2.3. Investigation of CBS Protein Specificity by HPLC

To assess the specificity of the purified CBS protein toward Hcy, 0.5 mg mL^−1^ of the protein was incubated with PBS (50 mM, pH 8.0) buffer containing 200 µM, 250 µM, and 300 µM of Cys, respectively. The reaction was carried out at room temperature (approximately 25 °C) for 5 min and then terminated with trichloroacetic acid, followed by derivatization using 7-fluorobenzo-2-oxa-1,3-diazole-4-sulphonate (SBD-F) as the derivatizing agent. The remaining Cys in the reaction mixture was quantified by high-performance liquid chromatography (HPLC), as detailed in a previous study [38].

### 2.3. Preparation and Detection of CBS/NPG/GCE Electrode

NPG was prepared by the corrosion of a 12K gold–silver alloy film (Au50Ag50 wt%, Sepp Leaf Products, New York, NY, USA) using concentrated nitric acid. The process was as follows: First, the gold–silver alloy film (100 nm thick) was cut into 6 mm × 6 mm pieces and immersed in approximately 50 mL of preheated concentrated nitric acid (40 °C) for 60 min. After the corrosion process was complete, the NPG was transferred to ultrapure water and washed to neutral pH. The resulting NPG film was subsequently coated onto the surface of the glassy carbon electrode (GCE) and 3 µL of 0.5% Nafion solution was added to cover the electrode surface, followed by drying and baking under a sodium lamp for 15 min to produce the NPG/GCE electrode.

The prepared NPG/GCE electrode served as the working electrode, with a platinum electrode as the auxiliary electrode and a saturated calomel electrode as the reference electrode, forming a three-electrode system, and the electrochemical measurements were conducted at room temperature using an electrochemical workstation (CHI 760E). The prepared NPG/GCE electrodes were subjected to cyclic voltammetry (CV) in 0.5 mM of diluted sulfuric acid at a sweep rate of 50 mV s^−1^. The effective catalytic area of the NPG/GCE electrode was characterized by the amount of oxide formed on its surface [39]. Then, the NPG/GCE electrode was washed with ultrapure water and transferred to PBS (50 mM, pH 8.0) for further CV testing at a scan rate of 50 mV s^−1^ to activate the electrode. According to Equation (1), the current density (j) can be determined with units of μA cm^−2^.

(1) $$j=\frac{I}{A}$$

where I is the current (μA) and A represents the effective catalytic area of the NPG/GCE electrode (cm^2^). All experiments in this study were conducted under anaerobic conditions, and the potentials in all experiments were referenced to a saturated calomel electrode.

For the fabrication of the cystathionine β-synthase-modified electrode (CBS/NPG/GCE electrode), the prepared NPG/GCE electrode was immersed in a purified CBS enzyme solution (diluted to 2 mg mL^−1^) and stored at 4 °C for 72 h to allow for enzyme immobilization. The CBS/NPG/GCE electrode was then rinsed with ultrapure water to remove any excess non-immobilized enzyme and stored at 4 °C for subsequent use. Different concentrations of Hcy were added into a PBS (50 mM, pH 8.0) buffer solution containing a fixed concentration of 200 µM of Cys, and the current responses corresponding to these varying concentrations of Hcy were measured using the amperometric i-t method with the CBS/NPG/GCE electrode developed in this study. The anti-interference capability of the CBS/NPG/GCE electrode was assessed by adding common interferents to the test buffer solution and comparing the current responses before and after the addition of the interferents. A spiked recovery experiment was conducted by mixing 1.5 mL of urine with 13.5 mL of PBS (50 mM, pH 8.0) to create a diluted urine sample, to which a fixed 200 µM of Cys and varying concentrations of Hcy were added, and Hcy was quantified using the CBS/NPG/GCE electrode to assess its practical applicability in real sample analysis.

## 3. Results and Discussion

### 3.1. Heterologous Expression and Activity Verification of the CBS Protein

#### 3.1.1. Construction of Recombinant Cystathionine β-Synthase

**Figure 1:**
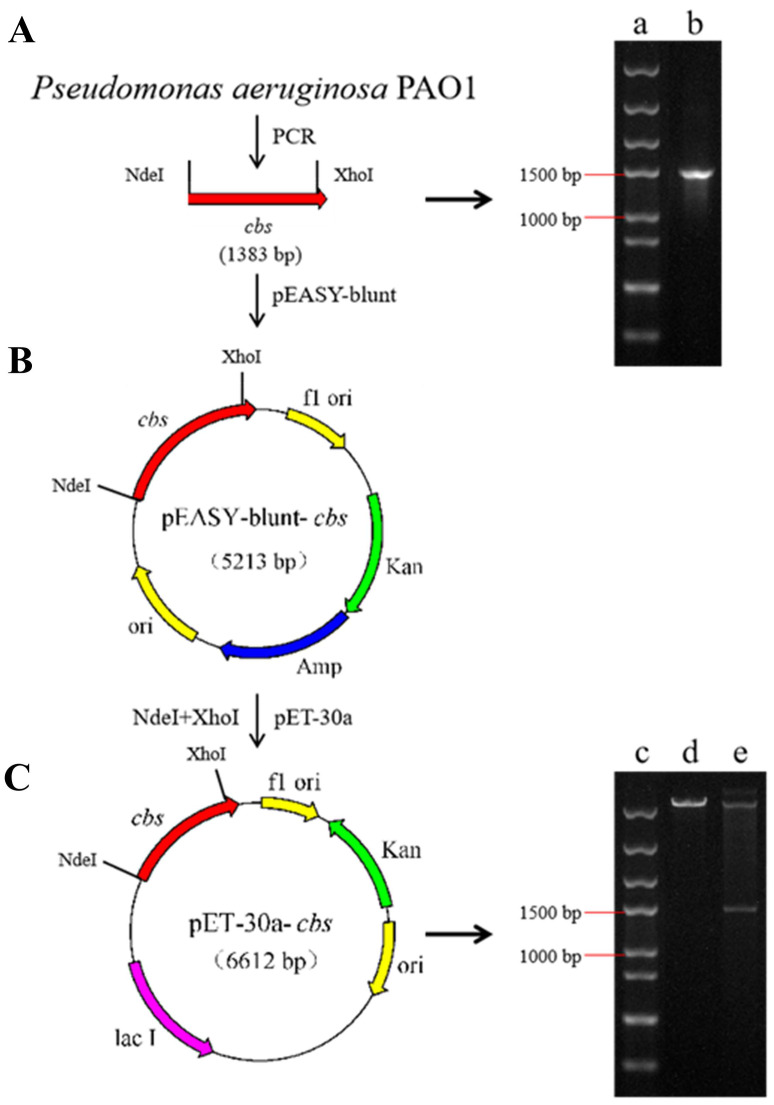
The schematic construction of recombinant expression vector (pET-30a-*cbs*) and electrophoresis images of PCR amplification and double digestion. (**A**) The PCR amplification and electrophoresis analysis for the target gene *cbs*. (**B**) The schematic construction of recombinant cloning plasmid (pEASY-blunt-*cbs*). (**C**) The schematic construction of recombinant expression vector (pET-30a-*cbs*). Lanes a and c show the DNA marker, lane b shows the PCR product of the *cbs* gene, and lanes d and e display the double digestion of the pET-30a and pET-30a-*cbs* plasmids, respectively.

To construct the recombinant expression vector, PCR amplification was carried out using the *Pseudomonas aeruginosa* PAO1 genome as the template, and the resulting PCR product was analyzed by electrophoresis on a 1% (*w*/*v*) agarose gel. As depicted in Figure 1A (b), the PCR product exhibited a band approximately 1500 bp in size, which closely matched the theoretical size of the target gene, *cbs* (1383 bp). Following ligation of the recovered *cbs* gene (Figure 1B), the recombinant cloning plasmid (pEASY-blunt-*cbs*) was introduced into *E. coli* DH5α cells for amplification of the target gene and double digested with NdeI and XhoI after successful sequencing verification. The resulting fragments, excised and purified from the gel, were ligated into the similarly double-digested pET-30a expression vector using T4 DNA ligase to construct the pET-30a-*cbs* recombinant expression plasmid, as shown in Figure 1C. Subsequently, the constructed plasmid was introduced into the host strain *E. coli* BL21 (DE3), and positive clones were selected through restriction enzyme digestion with NdeI and XhoI. As shown in Figure 1C (e), the plasmid extracted from the positive clone exhibited two bands after double digestion: one band, larger than 5000 bp, corresponding to the pET-30a plasmid, and the other, approximately 1500 bp in size, representing the *cbs* target gene (1383 bp), confirming the successful introduction of the *cbs* gene into the selected positive clone strain, which could be used for the heterologous expression of CBS protein.

#### 3.1.2. Enzymatic Properties of the CBS Protein

**Figure 2:**
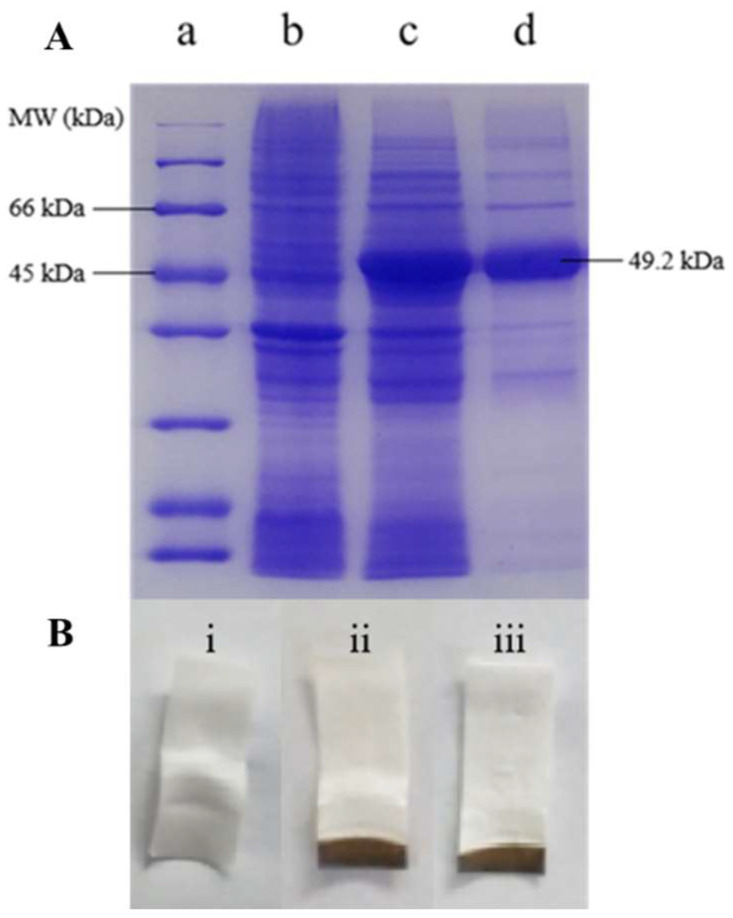
(**A**) The protein electrophoretogram of the crude enzyme solutions from induced *E. coli* BL21 (pET-30a) (lane b) and *E. coli* BL21 (pET-30a-*cbs*) (lane c), purified CBS enzyme solution (lane d), and protein marker (lane a). (**B**) Lead acetate test results for the crude enzyme solutions from *E. coli* BL21 (pET-30a) (i) and *E. coli* BL21 (pET-30a-*cbs*) (ii) and purified CBS enzyme solution (iii), reacting with 5 mM of Hcy + 5 mM of Cys.

To investigate the heterologous expression of CBS protein, the crude enzyme solutions from *E. coli* BL21 (pET-30a) and *E. coli* BL21 recombinant strain (pET-30a-*cbs*), as well as the purified CBS enzyme solution, were prepared and subsequently analyzed by protein electrophoresis. As shown in Figure 2A, lane c exhibited a significantly thickened protein band between 45 kDa and 66 kDa compared to lane b, which corresponds to the purified CBS protein (lane d) and aligns with the theoretical size of CBS protein (49.2 kDa), indicating the successful expression of the target protein in this experiment. Then, the enzymatic activity of purified CBS protein was tested using the lead acetate test strip method. Based on the principle of this method, the CBS protein reacts with the substrate (Hcy + Cys) to produce H_2_S, which is absorbed by the lead acetate test strip, resulting in the formation of lead sulfide and causing the white test paper to turn black. The reaction equation is as follows: $\mathrm{Pb}{\left({\mathrm{CH}}_{3}\mathrm{COO}\right)}_{2}+H_{2}S=\mathrm{PbS}+2CH_{3}\mathrm{COOH}$. Figure 2B (i) depicted that the crude enzyme solution induced by *E. coli* BL21 (pET-30a) did not react with the substrate to produce H_2_S, whereas the CBS protein obtained from the induced recombinant strain (pET-30a-*cbs*) could catalyze the substrate to generate H_2_S, as evidenced by the test strips (ii) and (iii) visibly turning black, confirming that the purified CBS protein in this experiment was active and suitable for subsequent experiments.

To assess whether the CBS protein, as the biomolecular component of the constructed sensor, can specifically detect Hcy, the Cys content in an enzyme reaction system devoid of Hcy was quantified using HPLC. As shown in Table 2, the remaining Cys content in the reaction system was nearly identical to the initially added amount, with the relative standard deviation (RSD) below 5%, indicating that the CBS protein scarcely reacts with Cys to produce H_2_S under the experimental conditions, consistent with the previous reports describing the negligible catalytic activity of CBS protein alone in generating H_2_S from Cys [28,29]. In addition, compared to the previously reported retention time of 2.4 min for Cys [38], the HPLC results of this study revealed a retention time of 4.1 min, which could be attributed to differences in column lengths. These findings confirm that the CBS protein predominantly catalyzes the production of H_2_S from the substrate combination of Hcy and Cys, enabling its application as a biorecognition element to specifically detect Hcy while effectively minimizing interference from Cys.

### 3.2. Construction and Characterization of the CBS/NPG/GCE Electrode

#### 3.2.1. The Detection Principle of the CBS/NPG/GCE Electrode

To construct the CBS protein-modified NPG/GCE electrode (CBS/NPG/GCE electrode), the prepared NPG/GCE electrode was immersed in the purified CBS enzyme solution, where the three-dimensional nanoporous structure of NPG provided abundant attachment sites for the enzyme molecules, allowing the CBS protein to be immobilized on the NPG surface through physical adsorption and gold-sulfur covalent bonding [39]. As depicted in Scheme 1B, the electrochemical reactions occurring on the CBS/NPG/GCE electrode result from the collaborative interaction between the exceptional electrochemical characteristics of NPG and the high catalytic specificity of the CBS protein. The proposed electrochemical mechanism for specific Hcy detection by the CBS/NPG/GCE electrode suggests that the CBS protein catalyzes the substrate (Hcy + Cys) to generate H_2_S, which is subsequently transformed by NPG into elemental sulfur and polysulfides through a two-electron transfer process, as reported in previous studies [40,41]. Therefore, the electrical signal response measured by the CBS/NPG/GCE electrode increases proportionally with the rising Hcy concentration, enabling the constructed electrode to specifically quantify Hcy.

**Figure 3:**
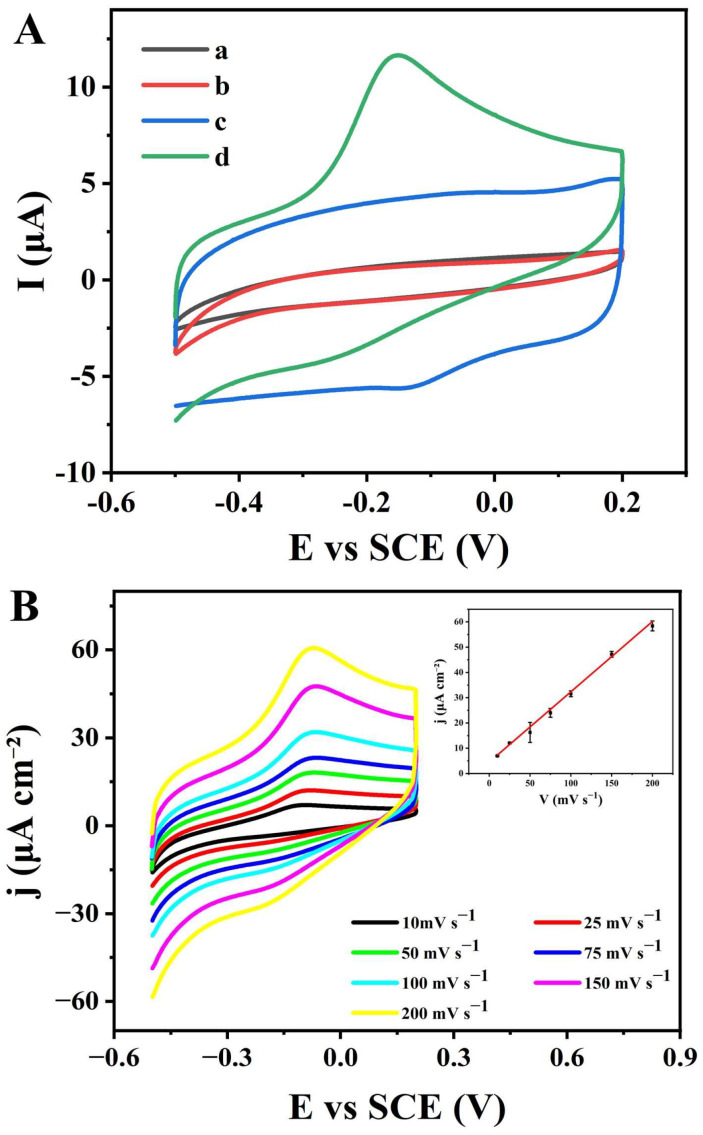
(**A**) The CV curves of GCE (a) and CBS/NPG/GCE (c) in PBS (50 mM, pH 8.0), as well as GCE (b) and CBS/NPG/GCE (d) in PBS (50 mM, pH 8.0) containing 200 µM of Hcy and 200 µM of Cys. (**B**) The CV curves of the CBS/NPG/GCE at different scan rates ranging from 10 to 200 mV s^−1^ in PBS (50 mM, pH 8.0) containing 150 μM of sodium sulfide. The inset profile shows the peak current density versus scan rate.

To assess the enhanced sensing capabilities of the CBS/NPG/GCE electrode, CV tests were initially performed on the bare GCE and CBS/NPG/GCE electrodes in a PBS (50 mM, pH 8.0) across a potential range of −0.5 V to +0.2 V at a scan rate of 50 mV s^−1^. As depicted in Figure 3A (a) and (c), compared with the uncoated GCE, the CV curve of the fabricated electrode exhibited a markedly improved overall current output, confirming that the distinctive catalytic features of NPG promoted electron mobility and boosted the signal performance of the GCE. In addition, the CV method was applied in a PBS (50 mM, pH 8.0) containing 200 µM of Hcy and 200 µM of Cys to further evaluate the potential of the CBS/NPG/GCE electrode for selective Hcy detection. As illustrated in Figure 3A (b) and (d), the bare GCE electrode failed to generate any catalytic current response within the potential range between −0.5 V and +0.2 V. In contrast, the CBS/NPG/GCE electrode exhibited a significantly stronger current signal in response to the substrate, with a distinct irreversible oxidation peak observed around −0.15 V, which aligned with the oxidation of sulfide catalyzed by NPG, as previously reported in the literature [42], indicating that H_2_S, the product of the CBS protein-catalyzed reaction, was oxidized on the CBS/NPG/GCE electrode surface, resulting in a pronounced peak current signal. These experimental results demonstrate that the CBS/NPG/GCE electrode developed in this study successfully enables the specific detection of Hcy by correlating the current response with changes in Hcy concentration, with sufficient Cys concentration ensured.

#### 3.2.2. Catalytic Kinetics of Sulfide at the CBS/NPG/GCE Electrode

Building on the synergistic interaction between CBS protein and NPG in the CBS/NPG/GCE electrode developed in this study, the CBS protein primarily facilitated the selective recognition of Hcy, while the observed oxidation current signal was predominantly attributed to the catalytic action of NPG on H_2_S. Thus, to further investigate the kinetic behavior of sulfide oxidation on the surface of the CBS/NPG/GCE bioelectrode, CV experiments were performed at varying scan rates ranging from 10 to 200 mV s^−1^ in a PBS (50 mM, pH 8.0) containing 150 μM of sodium sulfide, as shown in Figure 3B. As the scanning rate increased, the amplitude of the current density response for the sodium sulfide oxidation peak progressively rose, with only a single oxidation peak potential observed, confirming that the oxidation of sodium sulfide was an irreversible process. In addition, the oxidation peak current density exhibited a proportional increase with the scanning rate, demonstrating a strong linear relationship with a correlation coefficient of 0.996, indicating that the electrochemical oxidation of sulfide at the bioelectrode surface was a surface-controlled and irreversible process.

### 3.3. Hcy Detection Using the CBS/NPG/GCE Electrode

#### 3.3.1. The Applied Potential Optimization for Hcy Detection

**Figure 4:**
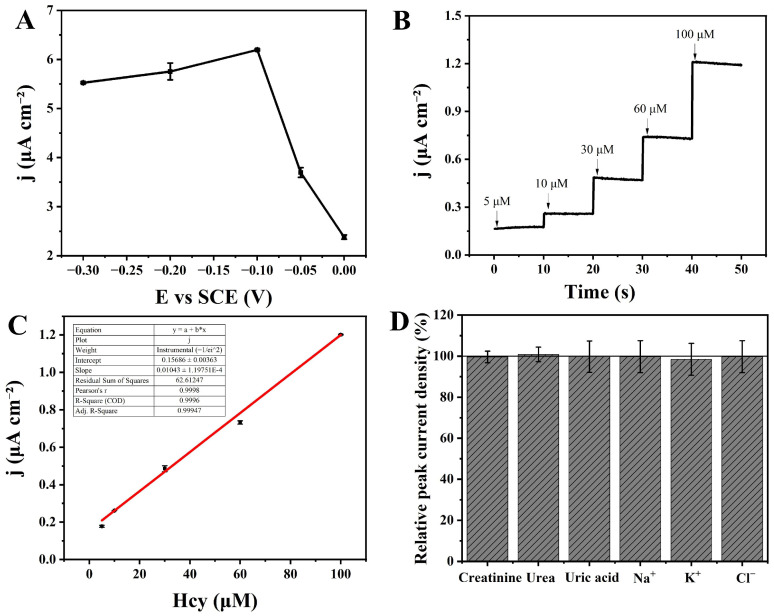
(**A**) Effect of the working potential on the current density response of the NPG/GCE in PBS (50 mM, pH 8.0) containing 100 µM sodium sulfide. (**B**) Amperometric i-t responses of the CBS/NPG/GCE upon successive additions of Hcy to PBS (50 mM, pH 8.0) containing 200 µM of Cys at the working potential of −0.1 V. (**C**) The linear relationship between the current density (μA cm^−2^) and the Hcy concentration (μM). (**D**) Effect of common compounds in urine on Hcy detection.

The amperometric i-t method necessitates selecting an optimal working potential to enable the highly sensitive detection of the target analyte. To optimize the applied potential for Hcy detection using the CBS/NPG/GCE electrode, the amperometric i-t responses of the NPG/GCE electrode were evaluated in a PBS (50 mM, pH 8.0) containing 100 µM sodium sulfide over a potential range of −0.3 V to 0.0 V, guided by the distinct oxidation peak current observed in Figure 3A. As illustrated in Figure 4A, the current density for sodium sulfide catalyzed by the NPG/GCE electrode steadily increased as the potential ranged from −0.3 to −0.1 V, after which the amperometric response began to decline with further increases in potential, reaching the maximum value at −0.1 V. Building on these results, the optimal applied potential for selective Hcy detection using the amperometric i-t method was set to −0.1 V for the subsequent experiments.

#### 3.3.2. The Detection of Hcy by Amperometric i-t Technique

Taking into account the result of applied potential optimization and the electrochemical behavior analysis for Hcy detection described above, the amperometric i-t method was employed to detect varying concentrations of Hcy in a PBS (50 mM, pH 8.0) containing 200 µM of Cys, with a fixed potential set at −0.1 V. As illustrated in Figure 4B, the steady-state current density detected by the CBS/NPG/GCE electrode progressively increased with the successive additions of Hcy at consistent time intervals, exhibiting a clear linear relationship within the concentration range of 5–100 µM, as shown in Figure 4C. The resulting linear equation was j (μA cm^−2^) = 0.01043 × C_Hcy_ (μM) + 0.15686 (R^2^ = 0.999), achieving a superior sensitivity of 10.43 μA mM^−1^ cm^−2^ and a detection limit as low as 1.31 μM (S/N = 3), highlighting its remarkable sensing capability for Hcy detection.

#### 3.3.3. Interference Resistance and Stability of the CBS/NPG/GCE Electrode

As a critical feature for the practical application of electrochemical sensors, interference resistance and stability were assessed by monitoring the amperometric i-t response of Hcy in the presence of common interference compounds typically found in urine test samples, like creatinine, urea, uric acid, Cl^−^, Na^+^, and K^+^. To evaluate anti-interference performance, 250 µM of interfering substances were individually introduced into a PBS (50 mM, pH 8.0) containing 200 µM of Cys and 50 µM of Hcy using the amperometric i-t technique at a constant potential of −0.1 V. As illustrated in Figure 4D, compared to the blank control, the amperometric i-t responses in the presence of interference substances revealed that all the interfering compounds caused minimal disruption, with interference levels well below 7.80%. Furthermore, the CBS protein in the bioelectrode did not catalyze Cys alone, effectively reducing the substantial interference from Cys, which shares similar properties with Hcy, in the sensor’s detection process. Thus, the CBS/NPG/GCE electrode can be regarded as exhibiting a highly selective response for Hcy detection, even in the presence of typical interferents like Cys, which were commonly found in medical samples.

#### 3.3.4. Real Sample Detection of Hcy

To assess the practical potential of the CBS/NPG/GCE electrode for selective Hcy detection, spiked recovery methods were conducted by detecting Hcy in diluted urine samples using the bioelectrode. For sample preparation, 1.5 mL of urine was first diluted with 13.5 mL of PBS (50 mM, pH 8.0), followed by the addition of 200 µM Cys and varying concentrations of Hcy, and the resulting samples were then analyzed using the amperometric i-t method. As shown in Table 3, the CBS/NPG/GCE electrode demonstrated excellent consistency between the spiked concentrations and the measured results, with recovery rates close to 100% and a relative standard deviation below 2.81%, indicating the high accuracy of the constructed bioelectrode for detecting Hcy in real sample analysis.

## 4. Conclusions

In this study, a heterologous expression system using *E. coli* BL21 (pET-30a-*cbs*) was developed, incorporating the *cbs* gene from *Pseudomonas aeruginosa* PAO1 as the target gene, resulting in the successful production of CBS protein with specific catalytic activity for substrates Hcy and Cys. Based on the specific substrate recognition ability of the CBS protein and the excellent electrochemical performance of NPG, the CBS/NPG/GCE sensor was successfully constructed for the specific detection of Hcy. The electrochemical mechanism demonstrated that the electrooxidation of sulfide, the catalytic product of Hcy and Cys, was a surface-controlled and irreversible process on the CBS/NPG/GCE electrode surface. In addition, the constructed sensor exhibited low detection limits, high sensitivity, and strong specificity in Hcy detection, and was successfully applied to real urine sample analysis, exhibiting excellent anti-interference ability and practical application potential while effectively minimizing the interfering impact of Cys. Building on the above results, the CBS/NPG/GCE sensor developed in this study achieved the rapid, efficient, and highly specific detection of Hcy, providing a novel approach for monitoring the urinalysis process. Future studies could focus on optimizing the CBS/NPG/GCE sensor for even lower detection limits and broader applicability by exploring different electrode modifications and additional enzyme variants. Furthermore, the integration of this sensor into point-of-care diagnostic devices for rapid on-site monitoring of Hcy levels could significantly enhance its clinical utility, particularly in personalized medicine and disease management.

## Figures and Tables

**Table 1:** PCR system and reaction conditions.

Reaction System	Reaction Temperature (°C)	Reaction Time		
The strain colony	95	10 min		
20 μM *cbs*-NdeI-F 2 μL	95	30 s	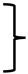	30 cycles
20 μM *cbs*-XhoI-R 2 μL	65	30 s		
FastPfu DNA Polymerase 4 μL	72	40 s		
5×FastPfu Buffer 40 μL	72	10 min		
10 mM dNTPs 4 μL	4	10 min		
ddH_2_O 148 μL				

**Table 2:** Determination of the remaining Cys content in the reaction system by HPLC.

Reaction System	Cys Added to the Reaction System (µM)	CBS Protein Volume (mg mL^−1^)	Cys Remaining in the Reaction System (µM)	RSD (%)
#1	200	0.5	202 ± 16	+1.00
#2	250	0.5	261.70 ± 0.15	+4.68
#3	300	0.5	299 ± 6	−0.20

**Table 3:** The spike recovery of Hcy detection in actual urine samples.

Sample	Spiked Hcy (µM)	Detected by CBS/NPG/GCE (µM)	Recovery Rate (%)	RSD (%)
#1	10	9.7 ± 0.6	97.19	−2.81
#2	30	29.8 ± 1.3	99.34	−0.66

## Data Availability

The data presented in this study are available on request from the corresponding author due to proprietary intellectual property restrictions.
